# Supplementary material for: An IL-1, IL-17, and IL-22 cytokine circuit controls vulvovaginal candidiasis independently of estrogen
Source: PLoS Pathog. 2026 May 7;22(5):e1014202. doi: 10.1371/journal.ppat.1014202 (PMC13167034; doi:10.1371/journal.ppat.1014202)
Supplement: S1 Fig — LDH levels in VLF (mU/mL) on day 3. Mean+SEM, analyzed by t-test with Welch’s correction. c. Blood glucose levels in serum (mg/dL). Mean+SEM, Welch’s correction. (DOCX) [file ppat.1014202.s001.docx]

**S1 Fig. Baseline LDH activity and blood glucose levels in Type 17-deficient mice.** **a, b.** LDH levels in VLF (mU/mL). Mean+SEM, analyzed by t-test with Welch’s correction. **c.** Blood glucose levels in serum (mg/dL).
