# Supplementary material for: An IL-1, IL-17, and IL-22 cytokine circuit controls vulvovaginal candidiasis independently of estrogen
Source: PLoS Pathog. 2026 May 7;22(5):e1014202. doi: 10.1371/journal.ppat.1014202 (PMC13167034; doi:10.1371/journal.ppat.1014202)
Supplement: S2 Fig — A-431 vulvar epithelial cells were treated with human IL-17 and/or IL-22 for the indicated times. Genes were assessed by qPCR relative to GAPDH and normalized to untreated control (0 minutes). Mean+SEM, analyzed by ANOVA and Tukey’s multiple comparisons test. (DOCX) [file ppat.1014202.s002.docx]

**S2 Fig**. **IL-17 and IL-22 synergy in human vaginal epithelial cells**. A-431 vulvar epithelial cells were treated with human IL-17 and/or IL-22 for the indicated times. Genes were assessed by qPCR relative to GAPDH and normalized to untreated control (0 minutes). Mean+SEM, analyzed by ANOVA and Tukey’s multiple comparisons test
