# Supplementary figures and images for: An IL-1, IL-17, and IL-22 cytokine circuit controls vulvovaginal candidiasis independently of estrogen
Source: PLoS Pathog. 2026 May 7;22(5):e1014202. doi: 10.1371/journal.ppat.1014202 (PMC13167034; doi:10.1371/journal.ppat.1014202)

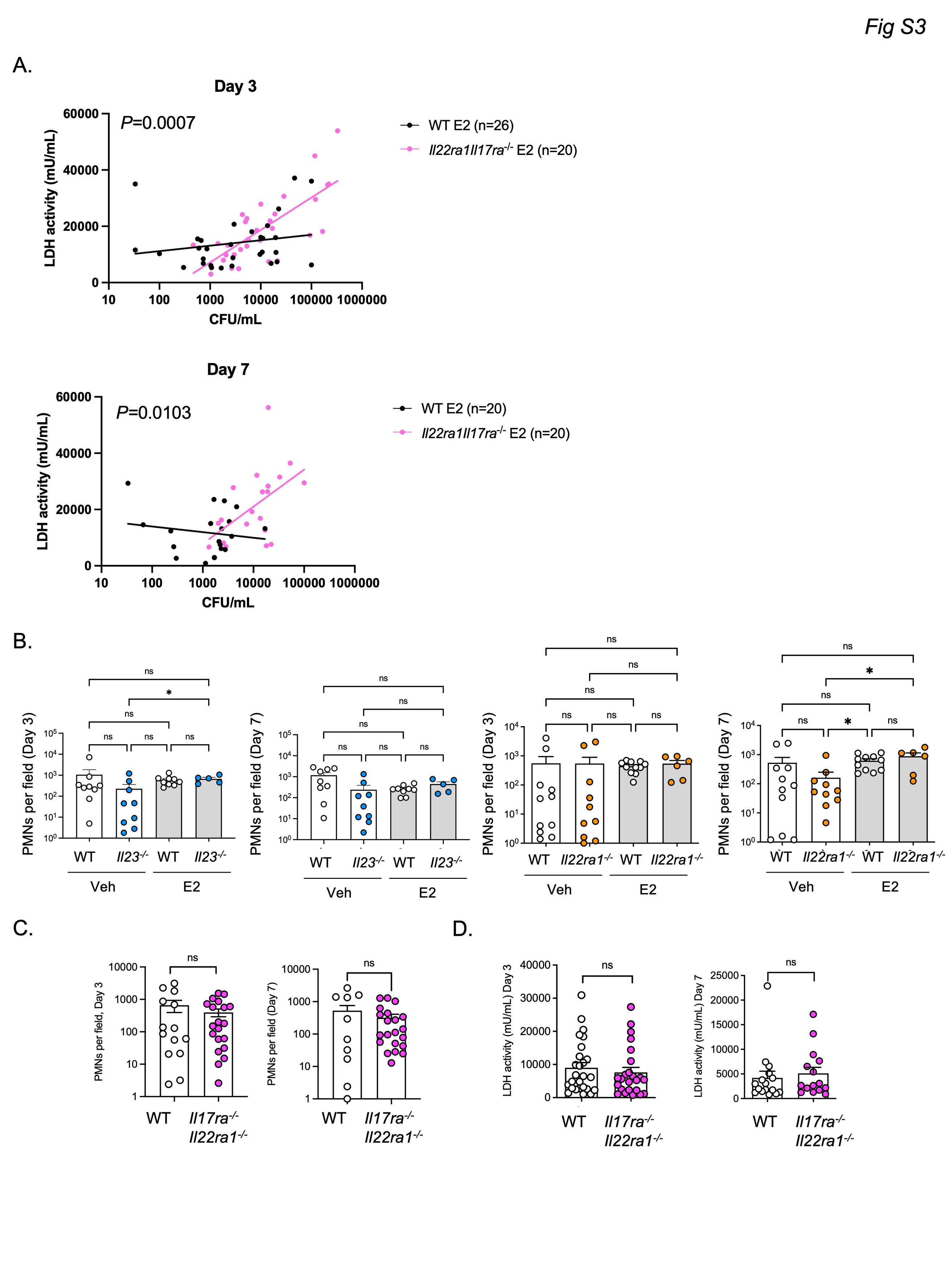

Supplement: S3 Fig — Correlation of LDH and CFU levels in estradiol (E2)-treated WT or Il17raIl22ra1-/- mice. Slopes compared by nonlinear regression. b. PMN counts in Il23-/- and Il22ra1-/- mice. PMNs in VLF determined by cytology, analyzed by two-tailed unpaired Student’s t-test with Welch’s correction. c. PMN counts on day 3 in SO treated Il17raIl22ra1-/- mice on days 3 and 7. Mean+SEM, analyzed by student’s t-test with Welch’s correction. d. LDH activity in VLF on day 3 and 7. Mean+SEM, analyzed by student’s t-test. (TIF) [file ppat.1014202.s003.tif]

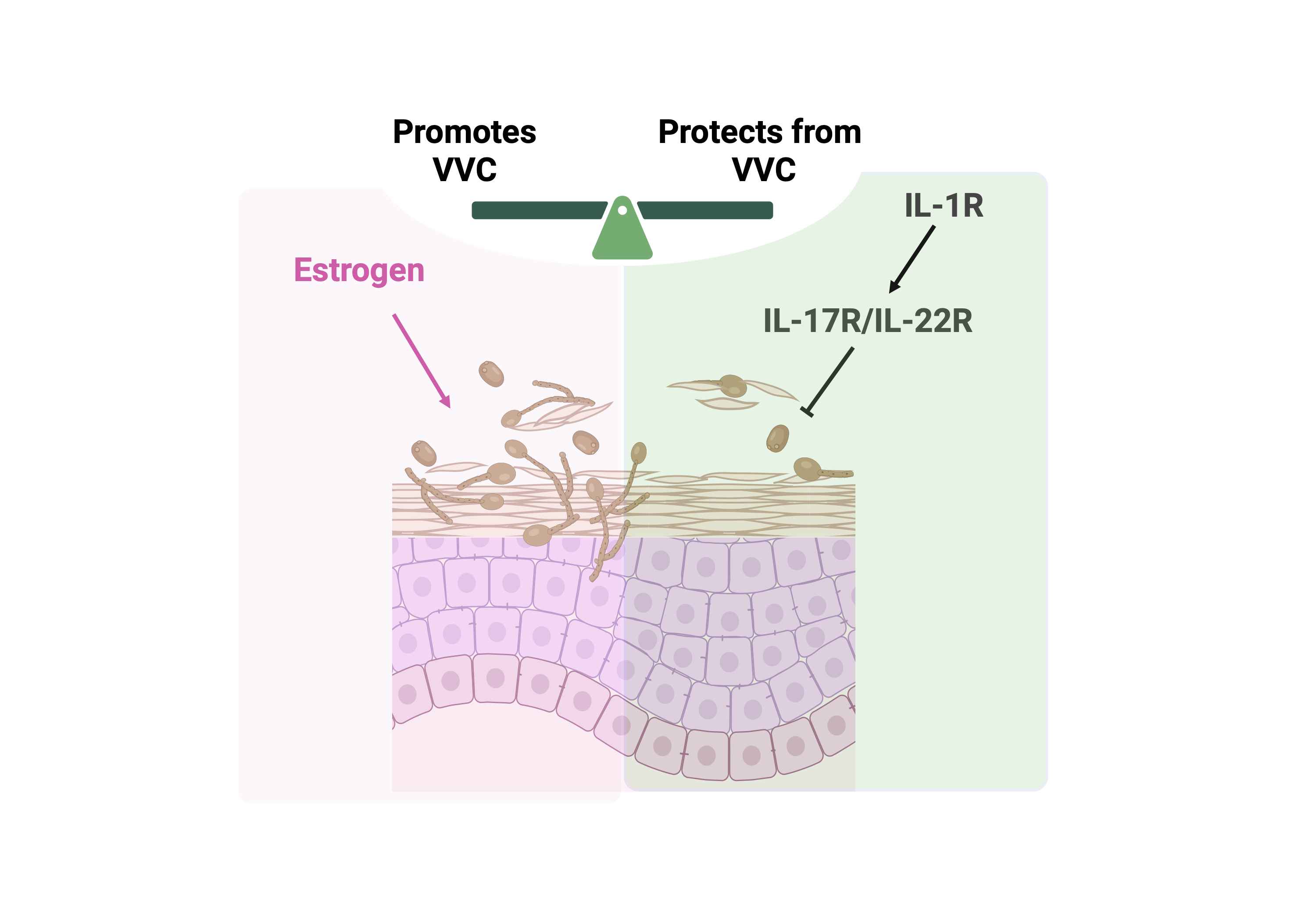

Supplement: S5 Fig — Created in BioRender. Gaffen, S. (2026) https://BioRender.com/unkcq15. (JPEG) [file ppat.1014202.s005.jpeg]

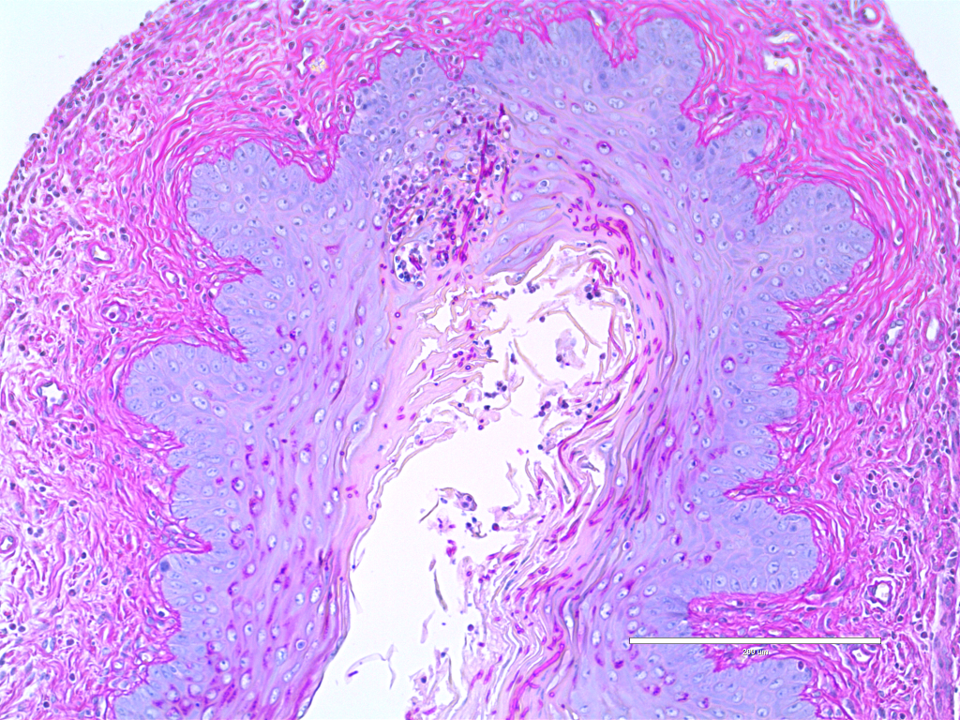

Supplement: S1 File — (ZIP) [file ppat.1014202.s011.zip › D3_DKO_E2_PAS_1.png]

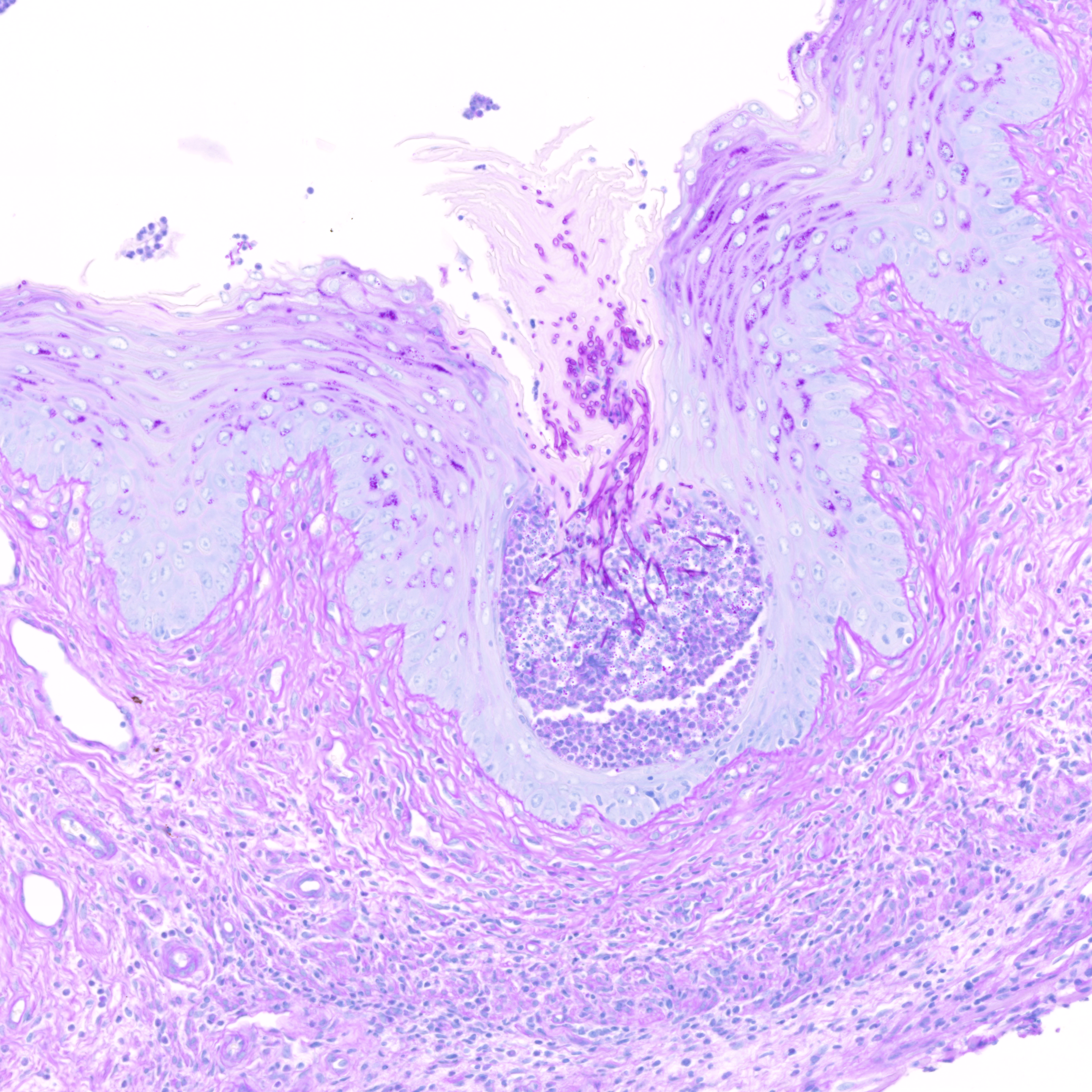

Supplement: S1 File — (ZIP) [file ppat.1014202.s011.zip › D3_DKO_E2_PAS_2.tif]

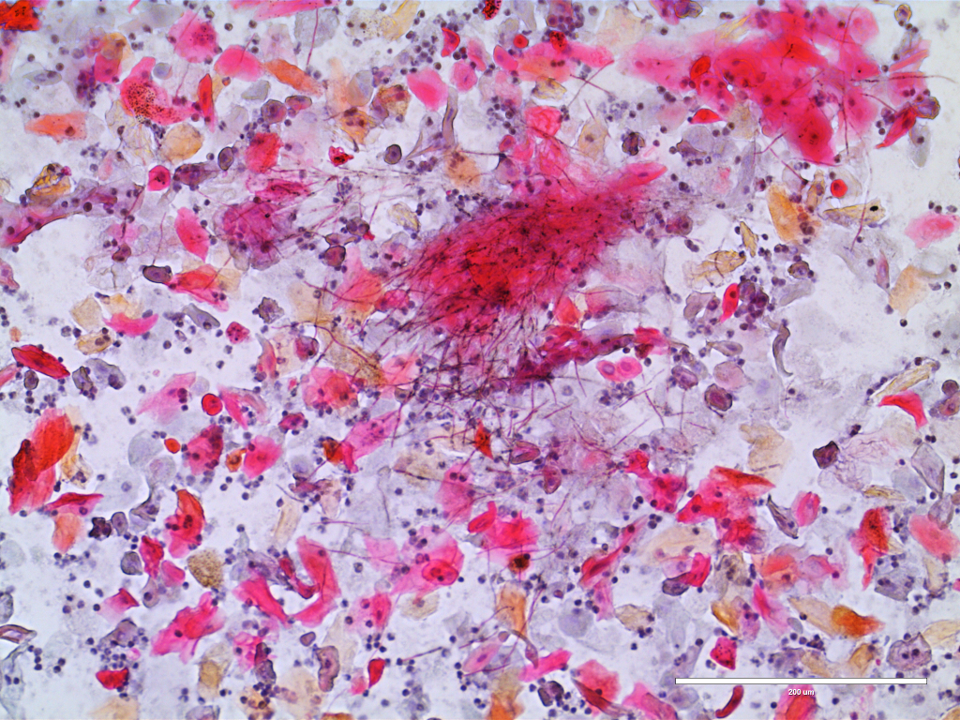

Supplement: S1 File — (ZIP) [file ppat.1014202.s011.zip › D7_DKO_E2_PAP.png]

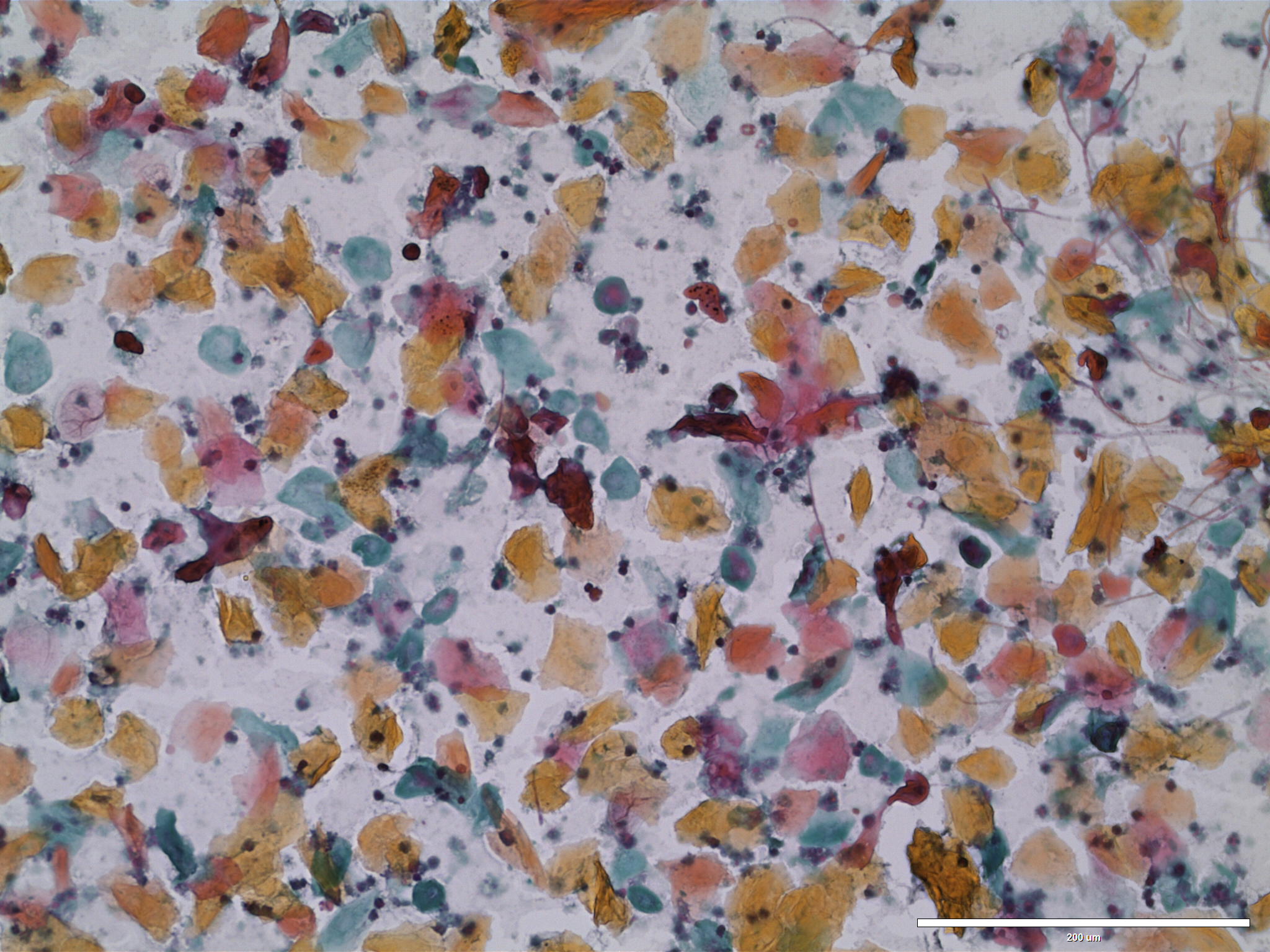

Supplement: S1 File — (ZIP) [file ppat.1014202.s011.zip › Striking image.tif]
