# Supplementary material for: An IL-1, IL-17, and IL-22 cytokine circuit controls vulvovaginal candidiasis independently of estrogen
Source: PLoS Pathog. 2026 May 7;22(5):e1014202. doi: 10.1371/journal.ppat.1014202 (PMC13167034; doi:10.1371/journal.ppat.1014202)
Supplement: S4 Fig — D: diestrus, M: metestrus, E: estrus, P: proestrus/estrus. b. Experimental setup. c. Representative PAP staining of day -1 VLF after E2 or P4 treatment. Created in BioRender. Cook, M. (2026) https://BioRender.com/7q9vqzi. d. Fungal loads in VLF on days 3 and 7 after sesame oil (Vehicle) (n = 7), estrogen (E2) (n = 6–7), or progesterone (P4) (n = 11–13) given on days -3 and 4 relative to infection. Mean ± geometric SD analyzed by two-tailed unpaired Mann-Whitney test. e. LDH activity in VLF on days 3 and 7 in P4-treated mice. t-test with Welch’s correction. (DOCX) [file ppat.1014202.s004.docx]

 **S4 Fig. Progesterone does not alter susceptibility to VVC**. **a**. Estrous cycle tracking by PAP staining of VLF over 12 days. D: diestrus, M: metestrus, E: estrus, P: proestrus/estrus. **b**. Experimental setup**. c**. Representative PAP staining of day -1 VLF after E2 or P4 treatment. **d.** Fungal loads in VLF on days 3 and 7 after sesame oil (Vehicle, n=7), estrogen (E2, n=6-7), or progesterone (P4, n=11-13) given on days -3 and 4 relative to infection. Mean ± geometric SD analyzed by two-tailed unpaired Mann-Whitney test. **e.** LDH activity in VLF on days 3 and 7 in P4-treated mice. t-test with Welch’s correction
