# Supplementary material for: An IL-1, IL-17, and IL-22 cytokine circuit controls vulvovaginal candidiasis independently of estrogen
Source: PLoS Pathog. 2026 May 7;22(5):e1014202. doi: 10.1371/journal.ppat.1014202 (PMC13167034; doi:10.1371/journal.ppat.1014202)
Supplement: S1 File — (ZIP) [file ppat.1014202.s011.zip › Striking image caption.docx]

Striking image captions


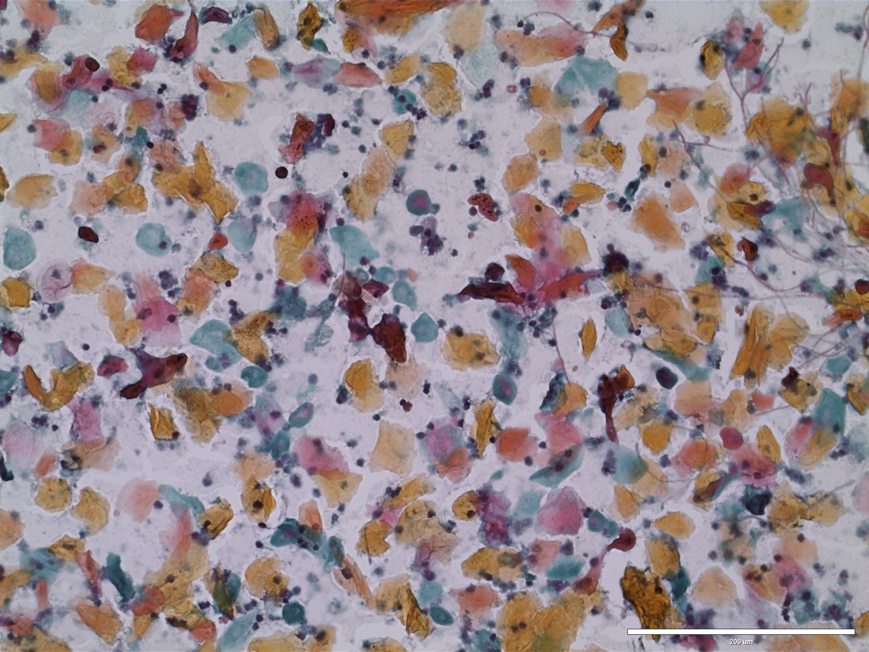


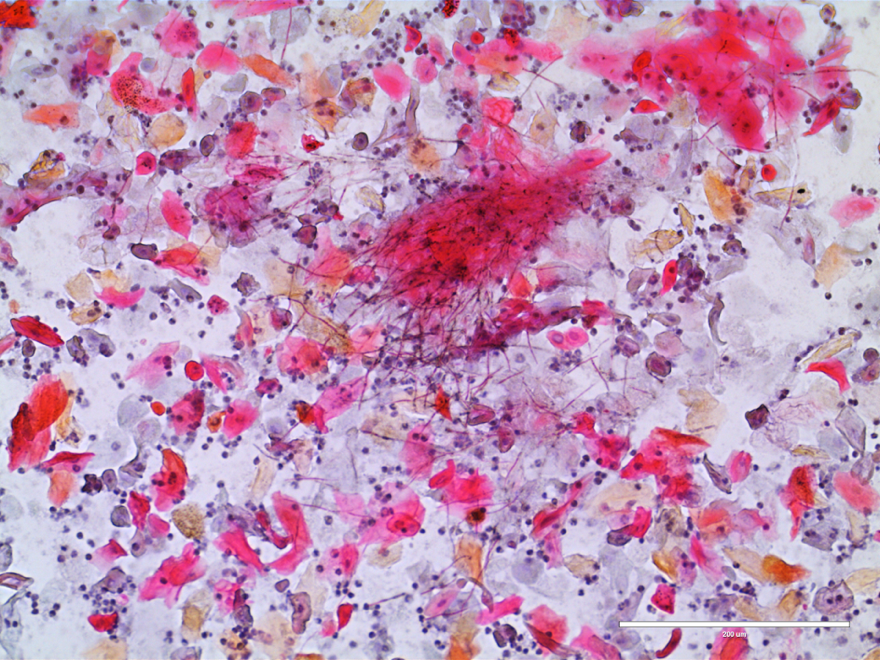


Vaginal lavage fluid from mice with vaginal *Candida albicans* infection was co-stained with Papanicolaou and hematoxylin. Nucleated and cornified vaginal epithelial cells (blue and pink/orange, respectively) are shed during the normal murine estrous cycle and during infection. *C. albicans*hyphae (pink stick-like cells) invade the epithelia and lead to neutrophil recruitment (small purple cells). This study identified host protective factors (IL-1R and IL-17R/IL-22R) that limit fungal infection in the vaginal tract. VLF sample is from an estrogen-treated *Il17raIl22ra1*^-/-^ mouse, day 7 post-infection.

Image credit: Melissa Cook


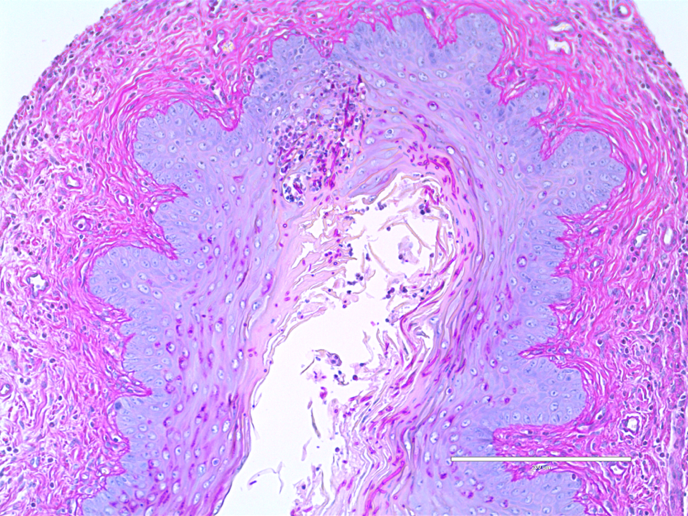


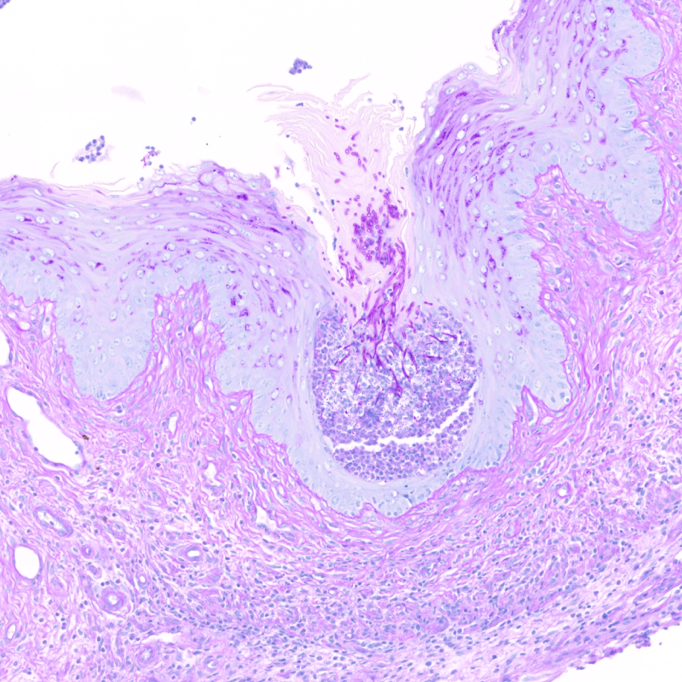


Mice were intravaginally infected with *Candida albicans* and vaginal tissue sections were co-stained with hematoxylin and Periodic Acid-Schiff (PAS). PAS brightly stains polysaccharides in fungal cell walls and identifies blastoconidia (small magenta cells) and hyphae (long magenta stick-like cells). *C. albicans* can invade the vaginal epithelium and recruit neutrophils (small purple cells). This study identified host protective factors (IL-1R and IL-17R/IL-22R) that limit fungal infection in the vaginal tract. This tissue sample is from an estrogen-treated *Il17raIl22ra1*^-/-^ mouse day 3 post-infection.

Image credit: Melissa Cook
